# Left Atrial Roof Enlargement Is a Distinct Feature of Heart Failure With Preserved Ejection Fraction

**DOI:** 10.1161/CIRCIMAGING.123.016424

**Published:** 2024-07-16

**Authors:** Sören J. Backhaus, Anastasia Nasopoulou, Torben Lange, Alexander Schulz, Ruben Evertz, Johannes T. Kowallick, Gerd Hasenfuß, Pablo Lamata, Andreas Schuster

**Affiliations:** 1 Department of Cardiology, Campus Kerckhoff of the Justus-Liebig-University Giessen, Kerckhoff-Clinic, Bad Nauheim, Germany (S.J.B.).; 2 German Center for Cardiovascular Research (DZHK), Partner Site Rhine-Main, Bad Nauheim, Germany (S.J.B.).; 3 Department of Biomedical Engineering, Division of Imaging Sciences and Biomedical Engineering, King’s College London, United Kingdom (A.N., P.L.).; 4 Department of Cardiology and Pneumology, University Medical Center Göttingen, Georg-August University, Germany (T.L., A. Schulz, R.E., G.H., A. Schuster).; 5 DZHK, Partner Site Lower Saxony, Germany (T.L., A. Schulz, R.E., J.T.K., G.H., A. Schuster).; 6 FORUM Radiology, Rosdorf, Germany (J.T.K.).; 7 FORUM Cardiology, Rosdorf, Germany (A. Schuster).; 8 School of Biomedical Engineering and Imaging Sciences, King’s College London, United Kingdom (A. Schuster).

**Keywords:** biomarkers, cardiomyopathies, hospitalization, models, statistical, phenotype

## Abstract

**BACKGROUND:**

It remains unknown to what extent intrinsic atrial cardiomyopathy or left ventricular diastolic dysfunction drive atrial remodeling and functional failure in heart failure with preserved ejection fraction (HFpEF). Computational 3-dimensional (3D) models fitted to cardiovascular magnetic resonance allow state-of-the-art anatomic and functional assessment, and we hypothesized to identify a phenotype linked to HFpEF.

**METHODS:**

Patients with exertional dyspnea and diastolic dysfunction on echocardiography (E/e′, >8) were prospectively recruited and classified as HFpEF or noncardiac dyspnea based on right heart catheterization. All patients underwent rest and exercise-stress right heart catheterization and cardiovascular magnetic resonance. Computational 3D anatomic left atrial (LA) models were generated based on short-axis cine sequences. A fully automated pipeline was developed to segment cardiovascular magnetic resonance images and build 3D statistical models of LA shape and find the 3D patterns discriminant between HFpEF and noncardiac dyspnea. In addition, atrial morphology and function were quantified by conventional volumetric analyses and deformation imaging. A clinical follow-up was conducted after 24 months for the evaluation of cardiovascular hospitalization.

**RESULTS:**

Beyond atrial size, the 3D LA models revealed roof dilation as the main feature found in masked HFpEF (diagnosed during exercise-stress only) preceding a pattern shift to overall atrial size in overt HFpEF (diagnosed at rest). Characteristics of the 3D model were integrated into the LA HFpEF shape score, a biomarker to characterize the gradual remodeling between noncardiac dyspnea and HFpEF. The LA HFpEF shape score was able to discriminate HFpEF (n=34) to noncardiac dyspnea (n=34; area under the curve, 0.81) and was associated with a risk for atrial fibrillation occurrence (hazard ratio, 1.02 [95% CI, 1.01–1.04]; *P*=0.003), as well as cardiovascular hospitalization (hazard ratio, 1.02 [95% CI, 1.00–1.04]; *P*=0.043).

**CONCLUSIONS:**

LA roof dilation is an early remodeling pattern in masked HFpEF advancing to overall LA enlargement in overt HFpEF. These distinct features predict the occurrence of atrial fibrillation and cardiovascular hospitalization.

**REGISTRATION:**

URL: https://www.clinicaltrials.gov; Unique identifier: NCT03260621.

**CLINICAL PERSPECTIVE**

Computational 3-dimensional left atrial anatomic postprocessing of cardiovascular magnetic resonance short-axis cine sequences allows identification of early phenotypic remodeling patterns in patients with heart failure with preserved ejection fraction (HFpEF), highlighting that atrial roof dilation is an early sign that precedes global left atrial dilatation in the progression of HFpEF, and provides initial evidence of its ability to predict risks of atrial fibrillation and cardiovascular hospitalization. Given new advances in the medical therapy of HFpEF, early identification of patients with HFpEF based on routinely acquired cine sequences may improve prognosis in HFpEF. As such, this shape signature could facilitate the early diagnosis of HFpEF and the development of risk prediction models. Identification of left atrial roof and pulmonary vein ostia dilatation may guide clinical decision-making in HFpEF with concomitant atrial fibrillation. Currently, the left atrial HFpEF shape score proposed is computed from a short-axis cardiovascular magnetic resonance acquisition; however, in the future, the methodology used can be extended to more widely used data sets based on clinical routine echocardiography.

**See Editorial by Patel and Shah**

To date, heart failure with preserved ejection fraction (HFpEF) accounts for >40% of patients with HF.^1^ Right heart catheterization is the reference standard for early diagnosis of diastolic dysfunction, which is defined by a threshold in pulmonary capillary wedge pressure (PCWP) at rest or during exercise-stress, respectively.^2^ However, first, right heart catheterization remains underused due to its invasive nature; second, an increase in PCWP reflects the consequence of cardiac remodeling rather than the underlying pathophysiological process. Meanwhile, exercise-stress echocardiography^3^ and cardiovascular magnetic resonance^4^ have been introduced to noninvasively unmask early functional—especially atrial—impairment during exercise-stress. Left atrial (LA) function reflects both left ventricular (LV) dysfunction and increased LV filling pressures, as well as intrinsic atrial dysfunction and stiffness,^5,6^ what might give an explanation as to why atrial functional quantification emerged with the highest accuracy for prediction of invasively proven HFpEF.^4^ The concept of atrial cardiomyopathy had formerly been introduced,^6^ and it is intriguing to speculate about the underlying atrial remodeling patterns in HFpEF causing loss of atrial function.

Advances in noninvasive imaging and computational postprocessing now allow for detailed analysis of cardiac remodeling^7^ and potentially mechanistic insights into remodeling caused by diastolic dysfunction. Moreover, cardiovascular computational modeling has now reached maturity, providing access to new biomarkers,^8,9^ while statistical models—atlases of the heart—have demonstrated the importance of shape in the diagnosis of cardiovascular disease.^10,11^ Specifically, comprehensive automated atrial remodeling assessment has previously demonstrated a predictive value for reoccurrence of atrial fibrillation (AF) following pulmonary vein (PV) ablation.^12^ These postprocessing analyses can be used on widely available routinely acquired balanced steady-state free-precession cine sequences at rest. Consequently, we sought to assesses LA remodeling in HFpEF, to identify specific remodeling features and to assess their clinical value.

## METHODS

### Study Population

The HFpEF-Stress trial (https://www.clinicaltrials.gov; unique identifier: NCT03260621)^4^ prospectively recruited 75 patients with exertional dyspnea (New York Heart Association class ≥II) and signs of diastolic dysfunction (E/e′, >8; ejection fraction, >50%) on echocardiography. Exclusion criteria comprised causes of dyspnea due to pulmonary (forced expiratory volume in 1 s, <80%) and cardiac (valvular, nonischemic, and ischemic heart disease) origin, as well as contraindications for cardiovascular magnetic resonance imaging and gadolinium-based contrast application.^13^ Patients underwent rest and exercise-stress right heart catheterization, echocardiography, and cardiovascular magnetic resonance imaging within 24 hours in stable sinus rhythm. Exercise-stress was performed on a bicycle ergometer in the supine position at 50 to 60 rpm aiming for an average heart rate between 100 and 110 bpm. HFpEF was defined according to current guideline recommendations^3^ based on PCWP assessment at rest (≥15 mm Hg, ie, overt HFpEF) or during exercise-stress (≥25 mm Hg, ie, masked HFpEF). In the absence of other findings, the remaining patients were classified as noncardiac dyspnea (NCD). A follow-up by telephone was conducted after 24 months additionally including a medical chart review. The data underlying the main findings are openly available at a FigShare repository: https://doi.org/10.6084/m9.figshare.26052298. The complete clinical images and data are available at the imaging database of the University Hospital Göttingen, and access will be granted to researchers who meet the criteria for access upon formal request. The study was conducted according to the principles of the Declaration of Helsinki and approved by the Local Ethics Committee at the University of Göttingen. All patients gave written informed consent before participation.

### Data Acquisition

#### Right Heart Catheterization

Fluoroscopy-assisted right heart catheterization was performed using a standard issue Swan-Ganz catheter introduced via access though the right internal jugular vein.^14^ After zeroing, right atrial, right ventricular, and pulmonary artery pressures, as well as PCWP, were assessed. Cardiac output was assessed by thermodilution based upon at least 3 consecutive valid measurements. Pulmonary artery pressure and PCWP, as well as cardiac output, were assessed for rest and exercise-stress testing.

#### Cardiovascular Magnetic Resonance

Patients were scanned on a 3.0T Magnetom Skyra MRI scanner (Siemens Healthcare, Erlangen, Germany).

##### Conventional

Conventional cardiovascular magnetic resonance imaging was performed using balanced steady-state free-precession cine sequences, which were acquired for long-axis (LAX) 2-, 3-, and 4-chamber views, as well as short-axis (SAX) orientations. Volumetric analyses were performed on SAX slices covering the entire heart. LV volumes were assessed in end systole (ES) and end diastole (ED). LA volumes were assessed at 3 instances: (1) at ES (reservoir); (2) at mid-diastole, the time point before atrial contraction (passive conduit); and (3) at ED (following active atrial contraction). Feature tracking deformation imaging was performed on LAX chamber view as appropriate using dedicated postprocessing software (2D CPA MR, Cardiac Performance Analysis; TomTec Imaging Systems, Unterschleissheim, Germany). Ventricular function was quantified by LV global longitudinal strain/global circumferential strain and right ventricular global longitudinal strain.^15^ Atrial function was quantified according to reservoir (Es), passive conduit (Ee), and active booster pump (Ea) function.^16–18^

##### Real Time

Real-time imaging was performed using balanced steady-state free-precession cine sequences on a strongly undersampled radial encoding scheme.^19^ Free-breathing cine imaging was acquired for 2- and 4-chamber views LAX, as well as SAX orientations. Volumetric analyses were evaluated in SAX orientations. LAX strain of all 4 cardiac chambers was assessed on LAX orientations as appropriate.^20^

#### Computational 3-Dimensional Atrial Modeling

##### Learning the Descriptors of LA 3-Dimensional Anatomy

A set of novel descriptors of LA 3-dimensional (3D) anatomy was learned from balanced steady-state free-precession SAX sequences covering the entire LA, in a similar fashion as reported previously^12^ (Figure S1).

First, a collection of smooth 3D computational meshes representative of the LA geometry was built by mesh personalization to each patient and cardiac phase. Briefly, a spherical template mesh for the atrial anatomy was fitted to each segmentation using image registration and warping methods described in the studies by Lamata et al.^21,22^ A consistent orientation across anatomies was enforced in this process by orienting meshes accordingly with 2 directions: the perpendicular direction to the SAX slices and the axis defined by the intersection between the SAX and the 3-chamber-view planes. LA meshes were built with smooth (cubic Hermite) interpolation functions, achieving a 3D reconstruction robust to acquisition or segmentation issues at the cost of losing anatomic detail.

Second, an unsupervised learning technique was used to learn the elementary patterns of LA shape variation observed in the cohort. Briefly, principal component analysis was applied to the collection of 3D LA meshes (n=204, 68 patients×3 phases) to generate the set of linearly independent modes of shape variation.^12^ As a result, each LA anatomy is described by a unique set of principal component analysis coefficients, each corresponding to and quantifying the contribution of each element within the set of modes of variation (ie, the new descriptors of LA anatomy change). This transformation provides us with a lower dimensional space (the set of modes of variation) that encodes each atrial anatomy instead of the original approach where each anatomy is described by the 3D coordinates of the 1.608 mesh nodes.

##### Learning the Signature of LA Anatomy That Associates With HFpEF

The LA 3D anatomic marker that maximizes the differences between the NCD and HFpEF groups was learned by an optimal linear combination of the principal component analysis modes. A supervised learning technique, linear discriminant analysis (LDA), was used and guided by the selection of features by the least absolute shrinkage and selection operator technique.^23,24^ The discriminatory performance of the LDA mode of shape variation was evaluated by a leave-one-out cross-validation technique and reported by the area under the curve (AUC) of the receiver operating characteristic curve.^12^

The LDA was first implemented in each independent phase of the cycle (ES, mid-diastole, or ED), finding a great similitude of the remodeling patterns and discriminatory performance across the 3 phases (Figures S2 and S3). Consequently, a single LDA bringing all phases together was performed to identify a unique score for HFpEF diagnosis.

#### Echocardiography

Echocardiography was performed for apical 2-, 3-, and 4-chamber views, as well as parasternal LAX (at rest only) and SAX orientations. Color Doppler imaging was performed for aortic, mitral, and tricuspid valve regurgitation assessment, continuous wave Doppler for aortic outflow and tricuspid regurgitation velocity assessment, as well as pulsed wave and tissue Doppler for assessment of septal and lateral E/e′, respectively. LA volume index was calculated based on a biplane approach from apical 2- and 4-chamber views.

### Statistical Analyses

Statistical calculations were performed using SPSS, version 27.0 (IBM, Armonk, NY), and MedCalc, version 20.111 (MedCalc Software bvba, Ostend, Belgium). The statistical shape model was built using in-house routines with Matlab (The MathWorks, Inc). Categorical variables are presented as frequencies and were compared using the χ^2^ test. Continuous variables are presented as medians with interquartile ranges and were compared using the nonparametric Mann-Whitney *U* test. Predictors for the presence of HFpEF were evaluated using Spearman rank correlation coefficients, Cox regression models, and area under the receiver operating characteristic curve analyses. AUC values were compared by the method proposed by DeLong et al.^25^

## RESULTS

### Study Population

Due to the novel diagnosis of specific cardiovascular diseases causative of dyspnea, 7 patients had to be excluded (coronary artery disease, n=4; amyloidosis, n=1; hypertrophic cardiomyopathy, n=1; and moderate aortic valve stenosis, n=1). The remaining 68 patients entering the final study population were classified according to PCWP into NCD (n=34) and HFpEF (n=34); the later was classified as overt HFpEF if diagnosed at rest (n=15) or masked HFpEF (n=19) if diagnosed during exercise-stress only.

Baseline characteristics have been reported previously^4^ (Table 1). Cardiovascular risk factors (*P*≥0.339) and symptom severity as estimated using the New York Heart Association classification (*P*=0.110) were distributed equally between HFpEF and NCD (*P*≥0.339). A history of AF was more common for HFpEF as opposed to patients with NCD (*P*=0.004).

**Table 1.**
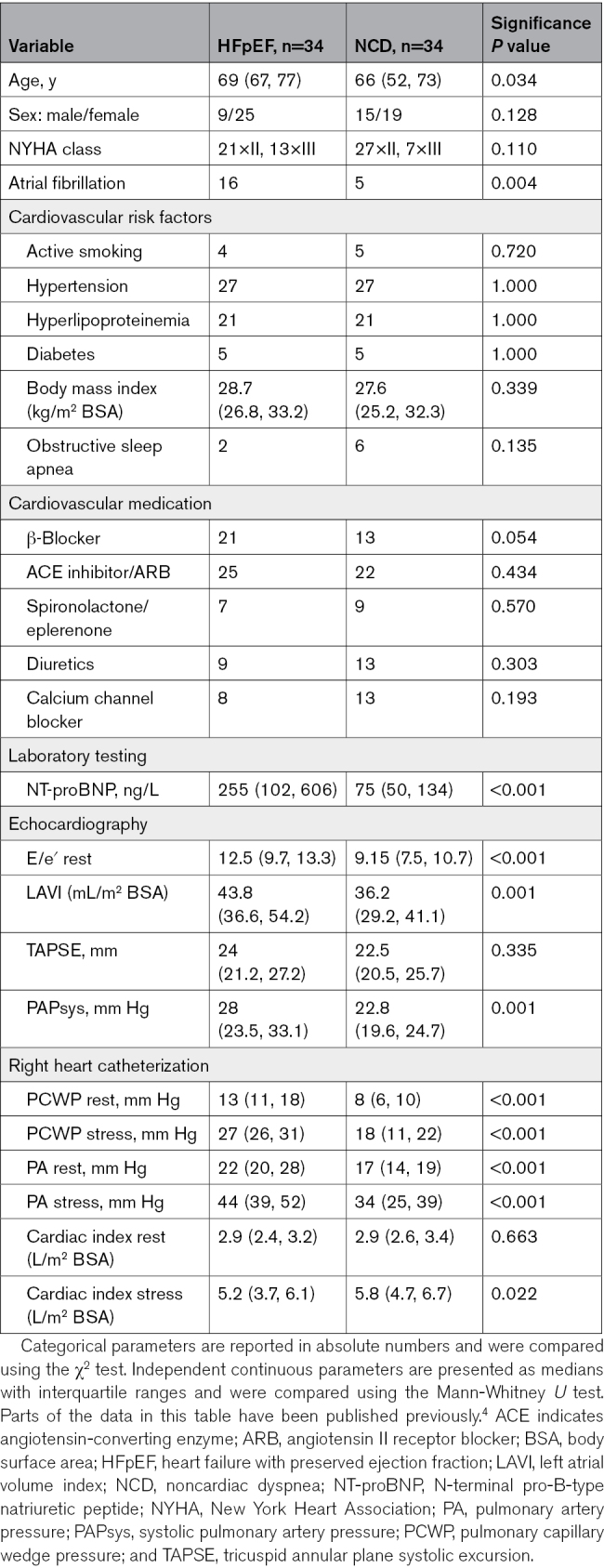
Patient Characteristics

### Computed LA Anatomy That Discriminates Between HFpEF and NCD

Modes 1, 3, and 7 were consistently selected by the least absolute shrinkage and selection operator feature selection in individual phases of the cardiac cycle (Figure S2). Mode 1 is the most discriminant and accounts for atrial size. Mode 3 accounts for a shift of the vertical axis of the atria in the septal to lateral direction. Mode 7 accounts for an atrial roof dilation (see the Supplemental Material for an illustration of the 10 first modes).

**Figure 1.**
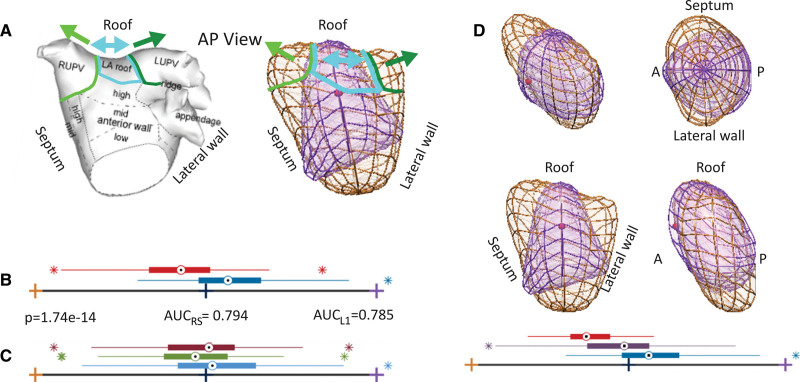
**The anatomic axes of remodeling of the left atria (LA) that discriminates heart failure with preserved ejection fraction (HFpEF) to noncardiac dyspnea. A**, Anterior-posterior (AP) view of a generic atria with its anatomic regions juxtaposed to the overlay between the shapes corresponding to the extreme HFpEF (orange color) and extreme noncardiac dyspnea (velvet color). Colored annotations identify the matching regions, and arrows guide the interpretation of the roof dilation (blue arrow) and pulmonary vein pulling (green arrows). **B**, Distribution of the HFpEF cohort (n=34, red box plot) and the noncardiac dyspnea cohort (n=34, blue box plot) in the axes of remodeling defined together with the significance of differences found (*P* value of unpaired *t* test) and the performance of the discriminant analysis by the area under the curve (AUC) in the resubstitution (AUC_RS_) and cross-validation (AUC_L1_) scenarios. **C**, Distribution of the atrial shapes at end diastole (blue box plot), end systole (green box plot), and mid-diastole (dark red box plot) in the axes of remodeling defined (entire sample, n=68). **D**, Four complementary views of the overlay of **A** (the red sphere indicates the direction identified in the intersection of the short-axis with the 3-chamber view, that is, toward the outflow track), together with the distribution of the atrial shapes in the overt HFpEF (n=15, red box plot), masked HFpEF (n=19, purple box plot), and noncardiac dyspnea (n=34, blue box plot) cohorts. Box plots do not indicate units since they represent *Z* scores between −3 and +3 SDs, between the orange and velvet extremes corresponding to shapes in **A** and **D**. LUPV indicates left upper pulmonary vein; and RUPV, right upper pulmonary vein.

**Figure 2.**
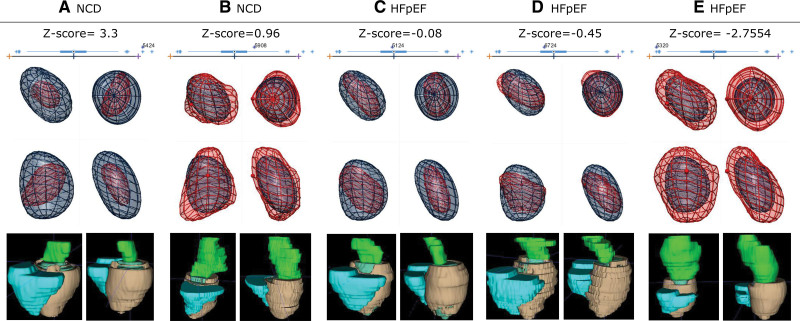
**Range of remodeling patterns in noncardiac dyspnea (NCD) and heart failure with preserved ejection fraction (HFpEF).** Exemplary 5 atrial anatomies (**A–E**) of cases with positive (ie, predictive of NCD) and negative (ie, predictive of HFpEF) scores accordingly to the axis of remodeling described in Figure 1. Each panel displays the same average atrial anatomy (blue mesh) overlaid to the case-specific mesh (red), using the same conventions as in Figure 1D. Note how case **B**, with such a large volume, still has a positive score and how cases **C** and **D** have a negative score despite their small size in comparison to conventional expectations **A** (small volume, positive score) and **E** (large volume, negative score).

These 3 modes (1, 3, and 7) were combined in a joint analysis with all phases together and led to the identification of a specific 3D remodeling pattern associated with risk for the presence of HFpEF. Figure 1 illustrates this pattern, characterized by a dilation of the atrial roof, as if it was pulled apart from the PV, and with a special emphasis from the right PV. As a result, all atrial shapes in our cohort (n=204, 68 patients×3 phases) are characterized by a LA HFpEF shape (LAHS) *Z* score, a new biomarker proposed to characterize the gradual remodeling happening between NCD and HFpEF and illustrated in Figure 2. A low score represents a high degree in atrial remodeling.

### Accuracy of LA Shape Reconstructions

The accuracy of the reconstructions is reported with the fitting error, that is, the distance between the surfaces of the 3D models to the contours of the LA blood pool that were used to generate them. This fitting error is 0.77±0.16 mm, with illustrative examples of the best and worst cases in Figure S4.

### LA Remodeling: Roof Dilation in Masked HFpEF but Size in Overt HFpEF

**Figure 3.**
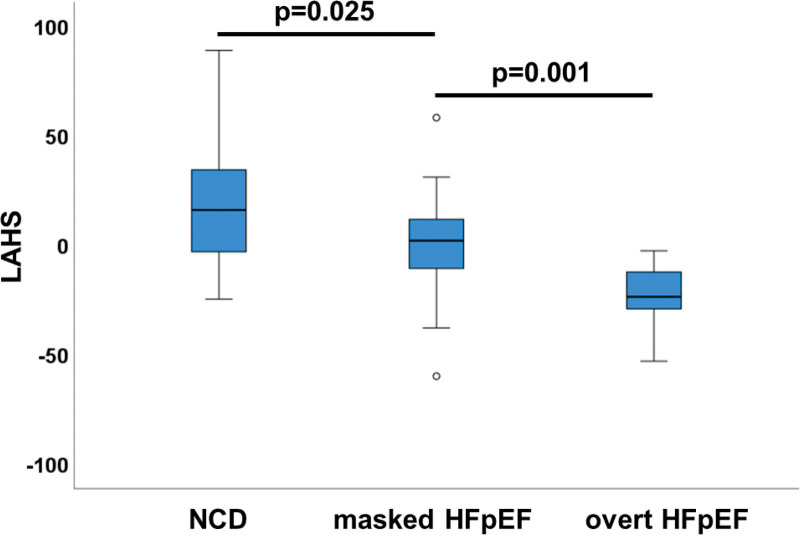
**Left atrial heart failure with preserved ejection fraction shape (LAHS) score according to the degree of diastolic dysfunction.** Noncardiac dyspnea (NCD), n=34; masked heart failure with preserved ejection fraction (HFpEF), n=19; overt HFpEF, n=15.

**Figure 4.**
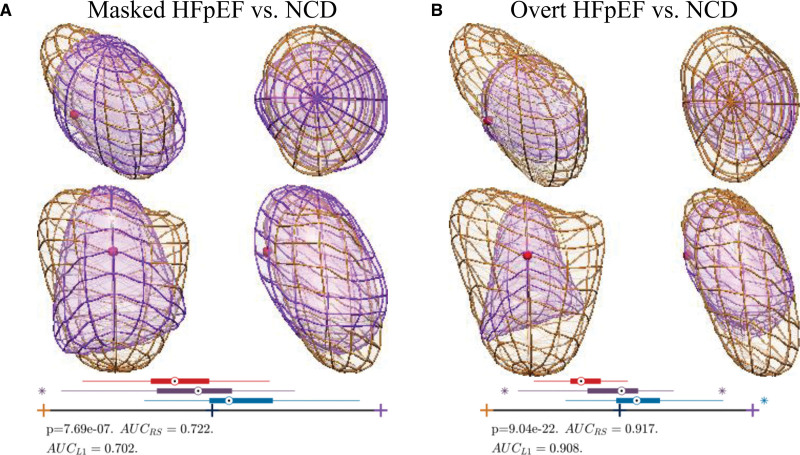
**The left atrial remodeling characteristic in masked and overt heart failure with preserved ejection fraction (HFpEF).** Four complementary views of the overlay between the shapes corresponding to the extreme HFpEF (orange color) and extreme noncardiac dyspnea (NCD; velvet color), as in Figure 1, with the distribution of the atrial shapes in the overt HFpEF (n=15, red box plot), masked HFpEF (n=19, purple box plot) and NCD (n=34, blue box plot) cohorts. The significance of differences and area under the curve (AUC) values at the bottom correspond to the comparison driving the analysis (ie, masked HFpEF vs NCD in **A** and overt HFpEF vs NCD in **B**).

Figure 3 demonstrates the increase in atrial remodeling according to the degree of diastolic dysfunction from NCD to masked HFpEF to overt HFpEF as appreciated from the LAHS score. While the degree of atrial remodeling is reflected in the LAHS score, we also tested the hypothesis that there are different dominant remodeling features in the 2 stages of the disease, masked and overt HFpEF. The weights of the 3 discriminant modes (1, 3, and 7) are optimized to maximize the differences first between NCD versus masked HFpEF, showing the dominance of the roof dilation, and second between NCD versus overt HFpEF, demonstrating the dominance of global LA enlargement (Figure 4).

Surprisingly, early-stage remodeling appreciated from roof dilatation is associated with mild decrease in ED volume (*P*=0.044) and ES volume (*P*=0.011) in conjunction with a statistical trend for improved passive conduit function (*P*=0.053) compared with nondilated atria. In contrast, late-stage remodeling appreciated from global dilatation results in deterioration of atrial morphology and function (*P*<0.001 except for LA Ea *P*=0.007; Table 2).

**Table 2.**
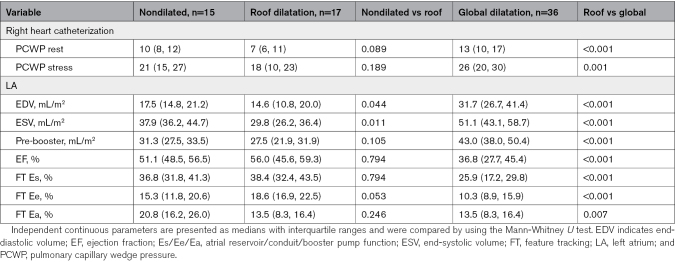
LA Function According to Remodeling Pattern

### LA Remodeling in the Context of Functional Alterations and Diagnostic Implications

Based on volumetric and deformation imaging, there were no differences in LV morphology and function comparing patients with HFpEF and NCD (*P*≥0.194). In contrast, LA volumes were increased in HFpEF compared with NCD (*P*<0.001). Furthermore, volumetric (*P*=0.003) and deformation imaging (*P*<0.001) revealed LA functional impairment in HFpEF (Table 3).

**Table 3.**
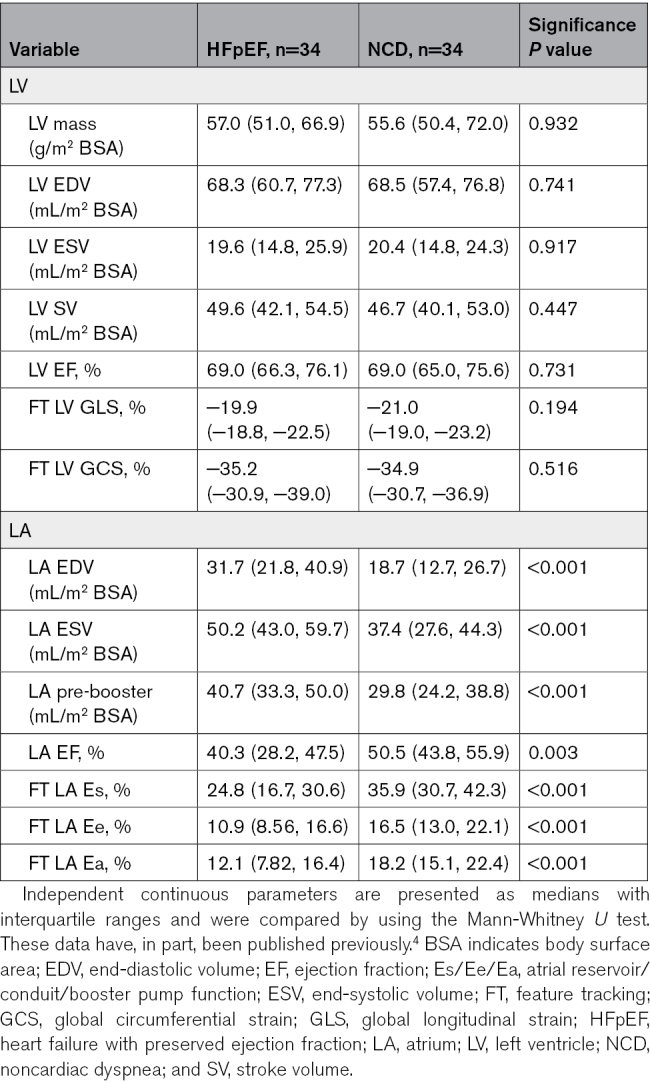
Cardiovascular Magnetic Resonance Imaging at Rest

LA morphological remodeling reported by a decrease in the LAHS score was associated with deterioration of LA phasic function (Es, r=0.61; Ee, r=0.53; Ea, r=0.48; *P*<0.001) at rest and impaired LA functional reserve from rest to exercise-stress (LA EF: rest, r=0.51; stress, r=0.45; LA LAX strain: rest, r=0.56; stress, r=0.57; *P*<0.001). The degree of specific 3D LA remodeling patterns was associated with the degree of congestion and postcapillary pulmonary hypertension demonstrated by correlations of LAHS with NT-proBNP (N-terminal pro-B-type natriuretic peptide; r=−0.53; *P*<0.001) and pulmonary artery pressure (rest: r=−0.53, *P*<0.001; stress: r=−0.48, *P*<0.001), as well as PCWP (rest: r=−0.63, *P*<0.001; stress: r=−0.52, *P*<0.001) at rest and during exercise-stress.

As a result, the LAHS score identified patients with HFpEF with high diagnostic accuracy (AUC, 0.81). In the discrimination between HFpEF and NCD, the LAHS score performed better than LA ejection fraction (AUC, 0.81 versus 0.72; *P*=0.094) with a strong statistical trend and equally good compared with dedicated deformation imaging total atrial strain Es (AUC, 0.81 versus 0.82; *P*=0.864). The LAHS score accurately predicted a history of AF (hazard ratio, 1.02 [95% CI, 1.01–1.04]; *P*=0.003; AUC to discriminate patients with/without AF, 0.74) and was associated with cardiovascular hospitalization during the 24 months of follow-up (hazard ratio, 1.02 [95% CI, 1.00–1.04]; *P*=0.043). All these results are obtained from the LAHS score at ED, the phase that showed slightly better performance than the other two.

## DISCUSSION

Based on the data collected in the HFpEF-Stress trial, this substudy identified 2 key features in LA anatomic remodeling in HFpEF: global enlargement and roof dilatation. Importantly, roof dilatation precedes atrial enlargement in masked HFpEF, the latter being the dominant pattern in overt HFpEF. Quantification of LA remodeling into a single LAHS score allows noninvasive discrimination between symptomatic patients with HFpEF and NCD and is associated with a history of AF and risk for cardiovascular hospitalization.

### Mechanisms Causing Atrial Roof Dilation

An increase in LV ED pressure originating from diastolic dysfunction translates to increased atrial pressures and subsequently increased PCWP, which is associated to symptom onset in HFpEF.^26^ A larger LA size is then expected to be caused by the larger pressures, but why does atrial roof dilation occur? The LA is irregularly shaped with varying wall thickness^27^; consequently, its anatomy is far more complex and irregular than LV morphology, while the LA walls are significantly thinner.^28^ Therefore, wall stress imposed by increased LV ED pressure may not be distributed equally within the thin walls of the atrium and these pressure gradients can have significant impact on regional deformation of the less stiff atrial walls as per the Laplace law.^29^ Data from a small previous study compared wall stress distribution and remodeling in 19 patients with AF before ablation^30^ and found a high variation of wall stress distribution patterns depending on atrial geometry. Peaks in wall stress were reported, among others, at the PV ostia (93%). Indeed, PV roots seem to be exposed to above-average stress during the cardiac cycle based on computational porcine models.^28^

Furthermore, an additional underlying mechanism introducing atrial wall stress is the traction imposed on the atrium by the movement of the mitral valve annulus plane. As the delicate PV ostia are the main structures directly attaching the heart without the nonfrictional support that the pericardial sac provides, they also are expected to provide resistance tractions to this motion, and, thus, stress concentrations are anticipated to occur in the PV ostia region due to atrioventricular plane displacements. In HFpEF, patients with an ejection fraction >60% show higher diastolic stiffness but increased LV contractility compared with patients with HFpEF in the ejection fraction range of 50% to 60%.^31^ The median LVEF in the present HFpEF population was 69%. Consequently, increased contractility and subsequent anulus plane movement may further contribute to wall stress on the PV roots and be a second factor contributing to larger LAHS scores in our study.

Our finding of an atrial roof dilation in HFpEF (Figure 2) can, therefore, be understood because of the aforementioned mechanisms. Our findings suggest an association of peak wall stress and dilatation pattern: the atrial roof and PV ostia may represent the predilection site of initial remodeling caused by localized peaks of wall stress distribution. Despite the fact that our 3D LA model is generated from a spheroidal template where the PV ostia are not explicitly included, the extensive remodeling in these structures is manifested through the observed atrial roof dilation.

### Roof Dilation as the Pattern That Precedes Global LA Enlargement

Intriguingly, an additional finding in our cohort was that in patients with an early stage of HFpEF as defined by PCWP increase during exercise only (ie, masked HFpEF), atrial roof/PV root dilatation emerged as the most discriminating characteristic with respect to NCD, achieving a moderate performance of an AUC of 0.70. This implies that stress-induced increases in wall stress may already promote changes in atrial remodeling before clinical onset of overt HFpEF. Following disease progress and PCWP increase at rest, that is, overt HFpEF, global atrial remodeling and overall atrial size take the lead in terms of diagnostic accuracy (with an AUC of 0.91; Figure 4). These findings suggest that roof dilation is an early and subtle manifestation already in masked HFpEF and that at a later stage of the disease (ie, overt HFpEF), the increment of LA size becomes a much stronger diagnostic marker. Indeed, comparing patients with nondilated atria to patients with atrial roof dilatation shows a mild but statistically significant decrease in overall atrial volumes with a strong statistical trend for improved diastolic LV filling/passive atria contraction forces. This finding may imply that these early remodeling patterns may to some degree compensate for early LV diastolic dysfunction. Following disease progression, overall atrial dilatation is then associated with complete atrial functional deterioration as seen in overt HFpEF.

### Roof Dilation as a Substrate for AF

Atrial size, stretch, and remodeling contribute to increased risk of AF occurrence and maintenance.^32^ Indeed, AF is a common finding in patients with HFpEF, and in the present sample, AF was significantly more frequent in HFpEF compared with patients with NCD.^33^ The PV roots are an important origin for AF burden and target for therapeutic ablation.^34^ Indeed, previous works have identified the LA roof dilation as the hallmark to predict AF recurrence.^35^ Consequently, it is intriguing to speculate about the link between PV root dilatation and AF occurrence in HFpEF. Indeed, the LAHS score displaying roof dilation was associated with a history of AF in the present sample. Furthermore, the LAHS score identified an elevated risk for cardiovascular hospitalization during the 24 months follow-up period. Indeed, prognostic performance of 3D mesh remodeling patterns had previously been established for LV remodeling by demonstrating superiority of LV 3D ES shape analyses over volume-based assessments following acute myocardial infarction.^36^

### Outlook

Future work involving more detailed images of the LA, ideally with full 3D coverage and isotropic spacing at acquisition, as well as larger patient cohorts, which will allow for more detailed anatomic models and analytical methods, respectively (as reported in 3), is required to refine the findings reported and to further ascertain the potential value of the 3D LA remodeling patterns for clinical diagnosis and risk prediction models.

### Study Limitations

The findings of this study should only be regarded as preliminary and partial due to the small sample size (n=68, with 34 subjects in each subgroup). The 3D anatomy of the LA body exhibits a large variability across subjects, and we can speculate that there will also be an equally large amount of variability of the response of 3D LA anatomy to the HFpEF burden, especially given the diverse etiology and still controversial definition of HFpEF. The coarse representation of the LA body as a spheroid, the use of a single LDA, and the use of cross-validation are thus key methodological choices to unravel the bulk 3D remodeling signatures with enough confidence. The similarity of findings across 3 different cardiac cycle phases brings additional confidence on the 3D remodeling patterns found. Future work involving a detailed shape analysis, with a 3D coverage in acquisition as the one reported in the study by Jia et al,^37^ is required to refine the early findings reported and ascertain the potential value of the 3D LA remodeling patterns for clinical diagnosis and risk prediction models.

### Conclusions

Computed 3D LA anatomy identifies roof dilation and atrial enlargement as the remodeling signature associated with HFpEF. Atrial roof dilatation precedes atrial enlargement in masked HFpEF, with overall dilatation/size being the dominant remodeling pattern in overt HFpEF. The degree of remodeling predicts the occurrence of AF and cardiovascular hospitalization.

## ARTICLE INFORMATION

### Sources of Funding

This study was supported by the German Centre for Cardiovascular Research, by the British Heart Foundation (PG/16/75/32383 and RE/18/2/34213) and by the Wellcome/Engineering and Physical Sciences Research Council Centre for Medical Engineering (WT203148/Z/16/Z). Dr Lamata was supported by a Wellcome Trust Senior Research Fellowship (209450/Z/17/Z).

### Disclosures

None.

### Supplemental Material

Figures S1–S4

## Supplementary Material
