# Supplementary material for: Left Atrial Roof Enlargement Is a Distinct Feature of Heart Failure With Preserved Ejection Fraction
Source: Circ Cardiovasc Imaging. 2024 Jul 16;17(7):e016424. doi: 10.1161/CIRCIMAGING.123.016424 (PMC11251503; doi:10.1161/CIRCIMAGING.123.016424)
Supplement: Supplementary file 1 [file hci-17-e016424-s001.pdf]

SUPPLEMENTAL MATERIAL

Supplemental Figures

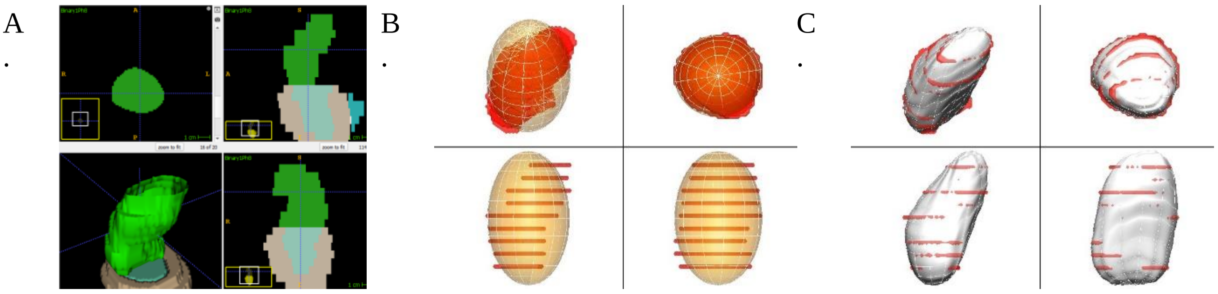

Figure S1: Illustration of the key steps in the mesh personalization pipeline.

Panel A: Segmentation of the atrial blood pool (green label) from the short axis MRI; Panel B: Initialization of the mesh personalization process, with the overlay of the segmentation (red disks) and the idealised spherical template of the left atrium (semi-transparent orange spherical mesh); Panel C: Result of the mesh personalization by image registration, where the patient-specific mesh (white mesh) has been adjusted to the contours of the segmentation (red disks).

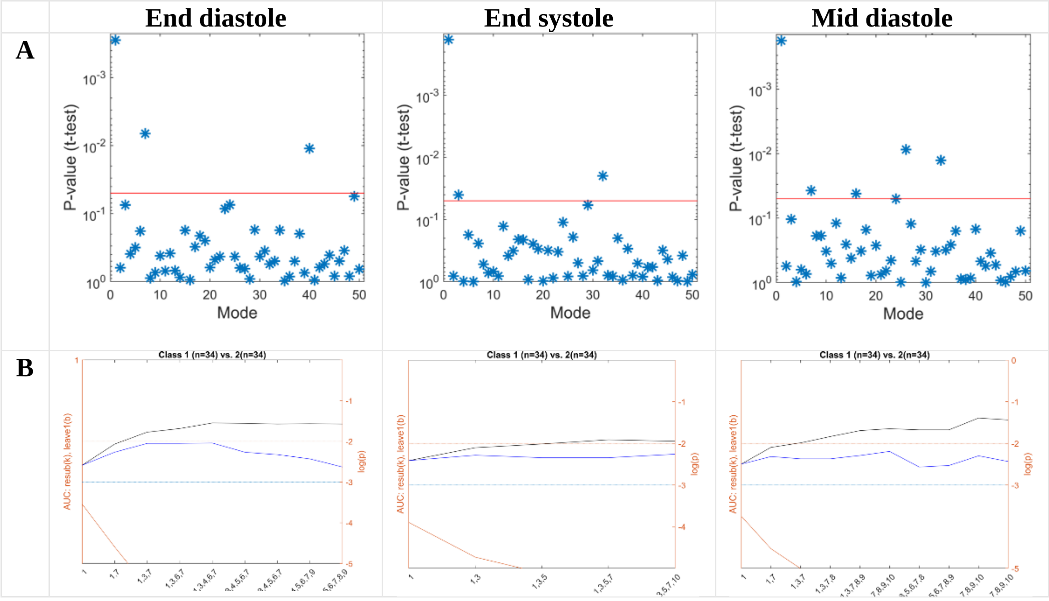

Figure S2: Identification of discriminant PCA modes in each cardiac phase. Panel A: Statistical significance between groups across the 50 first modes. Panel B: Gradual aggregation of PCA modes by LASSO feature selection, with resubstitution (black line) and cross-validation (blue line) discriminative performance (y axis, Area Under the receiver-operator Curve - AUC) as more modes are aggregated (x axis).

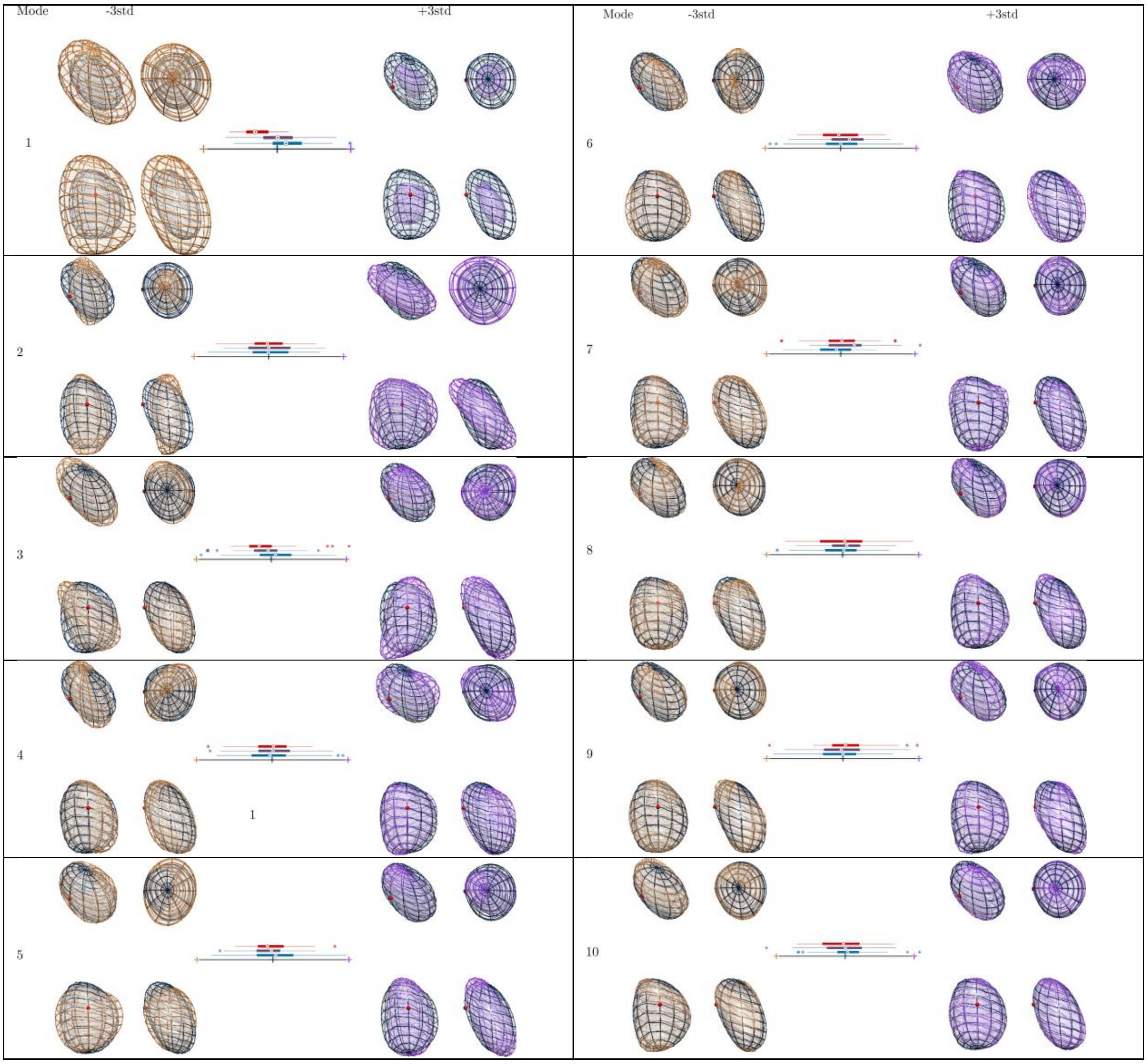

Figure S3: The first ten PCA modes of the statistical shape model, learned from the 68 subjects in their 3 phases (n=204 shapes). Each panel illustrates four complementary views of the overlay between the extreme shapes (orange and velvet shapes) with the average mesh (dark blue), and the box-plots of the distribution of each PCA mode in the overt HFpEF cohort (n=45 shapes, red box-plot), masked HFpEF cohort (n=57, purple box-plot) and NCD (n=102, blue box-plot).

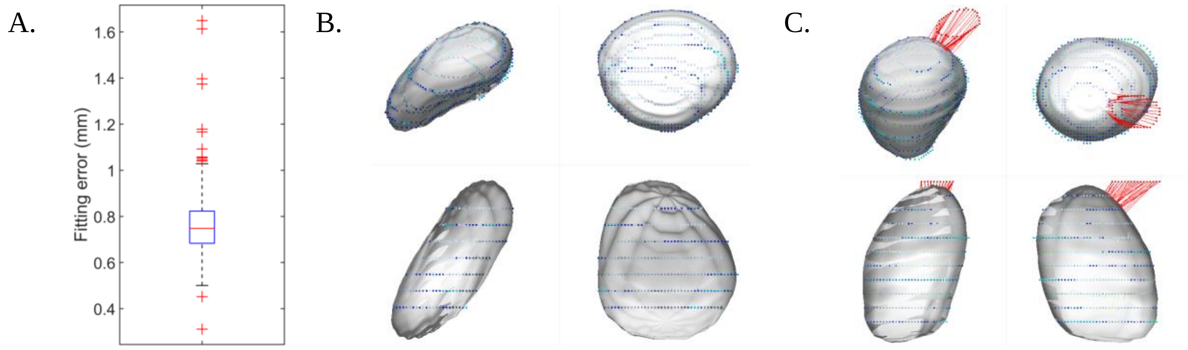

Figure S4: Accuracy of LA shape reconstructions, assessed by the fitting error.

Panel A: Box-plot of the fitting error in the set of 204 meshes reconstructed (68 cases at 3 individual phases). Panel B: Case with the smallest average fitting error, 0.31mm, illustrating four complementary views of the 3D model of the left atrium (white mesh) overlaid onto the contour points colour-coded by the fitting error (jet scale, with 0mm as blue and 1mm and higher as red). Panel C: Case with the largest average fitting error, 1.65mm, caused by a spurious extra segmentation slice, using the same illustration conventions as in panel B.
